# Comparison of Kato-Katz Thick Smear, Mini-FLOTAC, and Flukefinder for the Detection and Quantification of *Fasciola hepatica* Eggs in Artificially Spiked Human Stool

**DOI:** 10.4269/ajtmh.18-0988

**Published:** 2019-05-06

**Authors:** Daniel A. Zárate-Rendón, Johnny Vlaminck, Bruno Levecke, Andrea Briones-Montero, Peter Geldhof

**Affiliations:** 1 Laboratorio de Parasitología, Facultad de Zootecnia, Universiad Nacional Agraria La Molina, Lima, Peru;; 2 Laboratory of Parasitology, Faculty of Veterinary Medicine, University of Ghent, Merelbeke, Belgium

## Abstract

We compared the diagnostic performance of the standard method (Kato-Katz) with two recently developed methods (Mini-FLOTAC and Flukefinder) for the detection and quantification of *Fasciola hepatica* eggs in human stool. Uninfected human stool samples were artificially spiked with *F. hepatica* eggs to reach final concentrations of 14, 28, 41, or 96 eggs per gram of stool (epg). Only Flukefinder showed 100% sensitivity in all but the samples with the lowest concentration of eggs (14 epg), in which it had a sensitivity of 60%. Each of the methods underestimated the true fecal egg counts (FECs), Flukefinder resulting in the most biased egg counts (egg counts 0.18 times lower than the expected FECs). Only the Flukefinder resulted in more precise results (coefficient of variance < 30%) from FECs of 96 epg onward. The outcome of this study indicates that the Flukefinder is a useful alternative diagnostic method for human fascioliasis in stool.

Fascioliasis is caused by the flatworms *Fasciola hepatica* and *F. gigantica* and poses a major problem to both animal and public health.^1,2^ In humans, this parasitosis is an acknowledged (re-)emerging human disease in approximately 51 countries, its spread being closely linked to climatic conditions and affected by climatic change.^3^ The more important regions for human disease in the world are located in South America,^4^ with highly endemic areas in the Andean highlands.^2,3^ In recent decades, a variety of studies reported a hyperdendemic prevalence (> 10%) of human fascioliasis in these areas,^5,6^ accounting for approximately 17 million infected people and 180 million at risk of infection.^1^ Morbidity caused by fascioliasis is particularly pronounced in children,^7–9^ contributing to anemia and poor development.^10,11^ Despite the increased use of serological tests, the routine tests used to diagnose human fascioliasis in endemic rural areas are based on the microscopical demonstration and quantification of eggs in stool using coprological methods. One of the most widely used coprological methods is the Kato-Katz thick smear.^9^ This method has been extensively used to determine both prevalence and intensity of infection (based on the number of eggs per gram of stool; epg) in highly endemic areas.^12,13^ It is a cheap and simple method that has been standardized and recommended for a wider range of parasitic diseases in humans.^5^ However, an important limitation of this method is that it fails to detect infection of low intensity.^14^ Other coprological methods that hold promise as an alternative for Kato-Katz are the recently developed Mini-FLOTAC and Flukefinder test. Both methods have proven to have a high sensitivity for the diagnosis of fascioliasis in livestock,^15–17^ but they have not yet been fully validated for the diagnosis of human fascioliasis. In this study, we compared the diagnostic performance of the standard method (Kato-Katz) with the recently developed methods Mini-FLOTAC and Flukefinder for the detection and quantification of *F. hepatica* eggs in human stool samples.

The study protocol was approved by the Ethics Committee of the Universidad Peruana Cayetano Heredia (Code SIDISI: 17010) and Ghent University (Belgium (Code OZ 2017/0869). Negative stool samples were collected from children attending Institutional Day Care of the Universidad Nacional Agraria la Molina in Lima (Peru). This day care lies in an area which is nonendemic for fascioliasis. The collected samples were repeatedly examined by a modified rapid sedimentation test^5^ to confirm the absence of *F. hepatica* eggs. Confirmed *Fasciola-*negative stool samples were subsequently pooled and homogenized using wooden tongue depressors. The pooled sample was subsequently divided into four aliquots of 100 g each. This amount of stool allowed for repeated examination by all diagnostic methods. The aliquots were subsequently stored at 4°C for approximately 2 weeks until the experiments. Eggs of *F. hepatica* were obtained from bile and gall bladders collected from livers of naturally infected cattle that were slaughtered at an authorized abattoir in Lima. The eggs were purified and collected using the commercial Flukefinder method (Flukefinder) as described by the manufacturer’s instructions. The purified eggs were suspended in distilled water, and their concentration was determined by counting the number of eggs present in 10 aliquots of 10 μL each and subsequently calculating the arithmetic mean of these egg counts. Different volumes of the egg suspension were subsequently pipetted to the four aliquots of the pooled stool sample to obtain a final concentration of approximately 14, 28, 41, and 96 epg. A homogeneous distribution of the eggs was obtained in the stool by vigorously mixing the pooled stool using a metal plaster spatula.

The Kato-Katz thick smear (SterliTech, Kent, WA),^17^ Flukefinder (Flukefinder, Soda Springs, ID),^16^ and Mini-FLOTAC (Zinc Chloride flotation solution with a specific gravity of 1.30; University of Naples, Naples, Italy)^14^ methods were performed according to the manufacturer’s instructions. The fecal egg counts (FECs; expressed in epg) were obtained by multiplying the egg counts by 24 for Kato-Katz (0.417 g of stool is examined), 10 for Mini-FLOTAC (0.1 g of stool is examined = 2 mL of stool suspension which contains 2 g in a total volume 40 mL), and 0.5 for Flukefinder (2 g of stool is examined). Each method was performed 10 times on each of the four spiked samples. To avoid any systematic error in the results (e.g., first examining all aliquots with one particular method), the sequence of stool analysis was randomized. The sensitivity (proportion of samples in which eggs were found) was calculated across the three diagnostic methods and the four egg concentrations. The accuracy and precision of the FECs measured by the methods were based on the ratio observed over the expected FECs and the coefficient of variance (CV) (= SD/mean), respectively.

Overall, Flukefinder was the most sensitive method (90.0% [95% CI: 80.0; 97.5]), followed by Mini-FLOTAC (67.5 [95% CI: 52.5; 80.0]) and Kato-Katz (32.5% [95% CI: 17.5; 47.5]). As shown in Table 1, the sensitivity increased as a function of the number of eggs spiked, although this increase varied across the methods. The Flukefinder already reached a sensitivity of 100% for FECs of at least 28 epg, whereas for Mini-FLOTAC, this was only the case for the highest FECs (96 epg). Kato-Katz never reached a sensitivity of 100%, the sensitivity being 70% at most.

**Table 1:** The results of the spiking experiment using the Kato-Katz thick smear, Mini-FLOTAC, and Flukefinder on the samples artificially spiked with 14, 28, 41, or 96 *Fasciola hepatica* eggs per gram (epg)

Method	Replicate	Concentration of *F. hepatica* eggs per gram of stool (epg)			
		14	28	41	96
Kato-Katz	1	0	0	0	72
	2	0	24	0	120
	3	0	0	0	0
	4	0	48	0	0
	5	0	0	0	48
	6	0	24	48	168
	7	0	0	0	72
	8	24	24	0	24
	9	0	0	0	0
	10	0	0	0	24
Sensitivity (%)		10.0	40.0	10.0	70.0
Mean + SD		2.4 + 7.6	12 + 17.0	4.8 + 15.2	52.8 + 56.3
Accuracy (%)		17.1	42.9	11.7	55.0
CV (%)		44.3	39.6	129.7	102.4
Mini-FLOTAC	1	0	20	0	30
	2	10	20	0	30
	3	0	20	10	30
	4	20	10	0	20
	5	0	0	20	10
	6	10	0	20	30
	7	10	10	20	30
	8	0	10	10	10
	9	0	0	30	20
	10	0	0	30	20
Sensitivity (%)		40.0	60.0	70.0	100
Mean + SD		5 ± 7.1	9 ± 8.8	14 ± 11.7	23 ± 8.2
Accuracy (%)		35.7	32.1	34.1	24.9
CV (%)		19.8	27.2	34.4	34.3
Flukefinder	1	1	4.5	2.5	29.5
	2	0.5	9.5	1.5	28
	3	0	7	5	23
	4	1.5	6	5	32
	5	0	4	5	42.5
	6	1	11	2.5	30.5
	7	0.5	7.5	0.5	46
	8	0	6	3	24.5
	9	1.5	6.5	6	30
	10	0	17.5	6	37
Sensitivity (%)		60.0	100	100	100
Mean + SD		0.6 ± 0.6	7.95 ± 4.0	3.7 ± 1.9	32.3 ± 7.4
Accuracy (%)		4.3	28.4	9.0	33.6
CV (%)		14.3	14.0	21.6	22.1

CV = coefficient of variance; FEC = fecal egg count. Ten FEC replicates were performed for each method and the results are shown in epg. The accuracy equals the ratio observed over the expected FECs. CV = mean/SD.

The ratio of the observed epg over the expected FECs was < 1 for all the three methods, indicating that the methods underestimated FECs. The least biased FECs were obtained by Kato-Katz and Mini-FLOTAC, both methods resulting in FECs that on average were 0.31 times lower than the expected FECs. Fecal egg counts obtained by Flukefinder were 0.19 times lower. Table 1 illustrates the accuracy across the different levels of seeded eggs for the different methods. For Mini-FLOTAC, the percentage of accuracy in FECs remained relatively unchanged, ranging from 24% to 35.7%. This was in contrast to the other methods for which a wider range was observed (Kato-Katz: 17.1–55%; Flukefinder: 4.3–33.6%). Flukefinder was the most precise method of all, resulting in the lowest CV values across the different levels of seeded eggs. A CV not higher than 30% was obtained for FECs of 96 epg. The two other methods did not achieve a CV < 30%, although the CV of Mini-FLOTAC was 35% for the highest FECs (98 epg).

To the best of our knowledge, this is the first work that compares Kato-Katz, Mini-FLOTAC, and Flukefinder egg detection and quantification of *F. hepatica* eggs in artificially spiked human stool samples. Our results indicate that Flukefinder holds promise as an alternative to Kato-Katz for the diagnosis of human fascioliasis in stool. In contrast to the other methods, it shows a high sensitivity at low FECs (up to FECs of 28 epg) and it results in highly precise egg counts (CV < 30%) at FECs of 96 epg. The method underestimates FECs, but this was observed by all methods. Moreover, the Flukefinder could potentially also be used for the diagnosis of other major trematodiasis, such as paragonimiasis or schistosomiasis. Further research is now required to assess the clinical sensitivity and the user-friendliness of the Flukefinder under field conditions.
